# Queer in Chem: Q&A with Dr Jovan Dragelj

**DOI:** 10.1038/s42004-023-00969-4

**Published:** 2023-09-30

## Abstract

Dr Jovan Dragelj completed his undergraduate and Master’s studies in chemistry in Belgrade, Serbia, after which he worked as a chemistry teacher and researcher at the University of Belgrade. In 2019, he earned his PhD in computational chemistry from Freie Universität Berlin and then pursued postdoctoral studies at Technische Universität Berlin. His research during this period spanned diverse areas, from non-covalent interactions to biocatalysis, with a major focus on studying cytochrome c oxidase and hydrogenase enzymes through multiscale modeling approaches.

**Figure:**
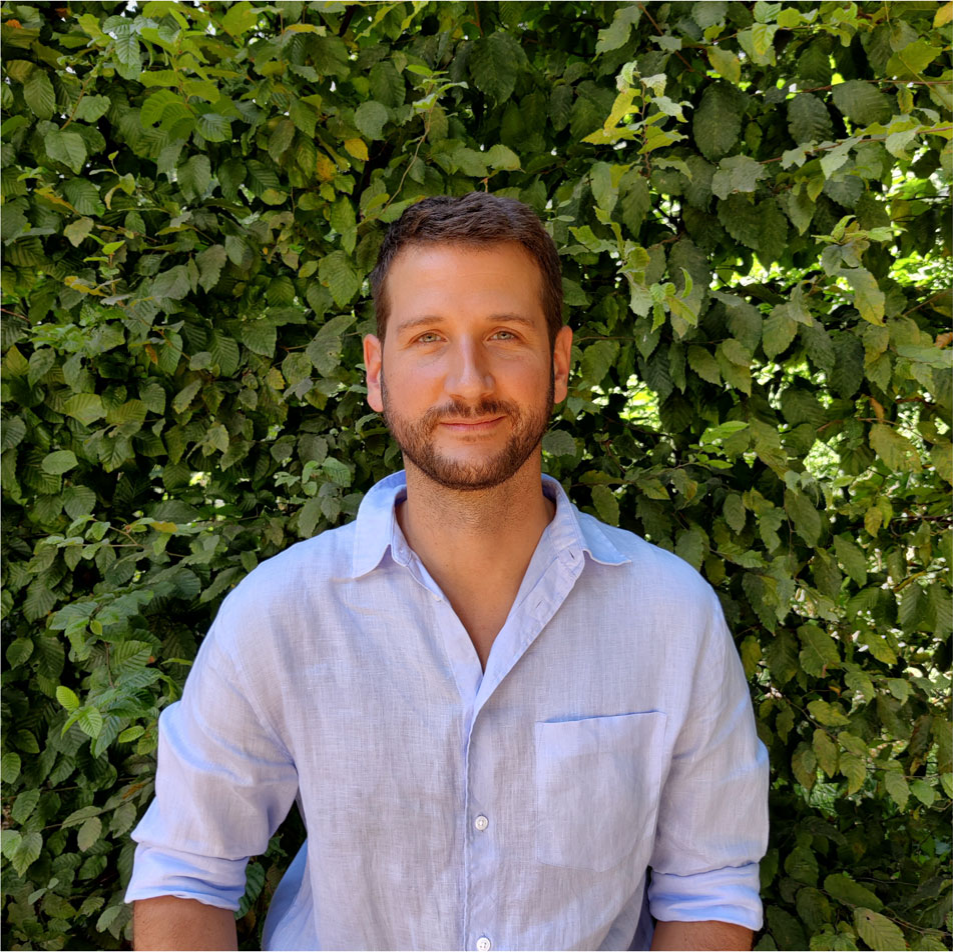
Jovan Dragelj

In recent years, Jovan has shifted his focus towards biomedical research, studying topics such as cell divisome and COVID-19. In 2023, he moved into the biotech industry, joining HotSpot Therapeutics, where he currently works on computational drug discovery. In 2019, alongside a group of like-minded individuals, Jovan co-founded LGBTQ STEM Berlin. This initiative is dedicated to promoting visibility and representation of LGBTQ+ individuals in STEM fields (Science, Technology, Engineering, and Mathematics).

Why did you choose to be a scientist?

I chose to become a scientist due to my innate curiosity. I’m continually intrigued by the reasons behind how the mechanisms behind things work—the thrill of discovery. When you are constantly questioning, you are constantly learning. It is not just a career but a fulfilling learning journey. Furthermore, I’m driven by the potential of scientific work to impact society positively and to contribute to our collective knowledge. This has a fascinating paradoxical effect—the more I learn, the more I realize how little we truly understand about the vast complexity of the world around us.

What scientific development are you currently most excited about?

I’m very excited about the convergence of artificial intelligence with various scientific domains like chemistry, biology, and physics. The idea of using advanced algorithms and computational models to simulate and predict complex scientific phenomena is truly captivating. We are in a digital era of science, often termed as ‘virtual science’ and it offers great potential in expediting discoveries and innovation. The power to analyze big data and combine it with in-silico experiments, and the speed at which we can now generate insights, is remarkable. It’s fascinating to contemplate how much further we can fuel scientific progress in this manner.

What direction do you think your research field should go in?

I believe computational chemistry (and other computational sciences) can contribute to significant advancements in two crucial sectors: life sciences and materials sciences. In life sciences, computational models are already employed to tackle health issues, potentially leading to treatments for various diseases. The power of predictive analysis and molecular simulations could pave the way for personalized medicine, and in the broader context, for significant advancements in global health.

On the other hand, in materials science, computational tools can contribute to addressing the climate crisis. It can aid in the development of new materials or optimize existing ones for renewable energy sources, carbon capture, and storage technologies.

How does your queer or trans identity intersect with your identity as a scientist?

As an active advocate for LGBTQ+ visibility in STEM, my queer identity significantly intertwines with my scientific one. Being openly gay in the scientific community not only affirms my personal identity, but it also adds a unique perspective in my professional environment. I firmly believe that diversity, including diversity of thought, from people with different backgrounds, propels scientific progress forward. I consciously choose not to separate these aspects of my identity, as I deem it crucial for promoting LGBTQ+ representation in the scientific community.

Why do you think it is important to feel comfortable enough to bring your whole self to work?

Feeling comfortable to bring your whole self to work, especially from an LGBTQ+ perspective, is essential. Being true to one’s identity eliminates the burden of constant concealment, which also frees mental energy for productivity. A safe environment fosters creativity and innovation, enhancing both individual and professional success. Moreover, an inclusive culture where individuals feel valued contributes to job satisfaction, employee engagement, and most importantly, overall mental health and well-being of those individuals. It is important to cultivate workplaces where everyone, including the LGBTQ+ individuals, can fully express themselves. Here I speak with some personal experience and there are articles published on this as well^1–5^.

Have you faced any challenging situations in your professional life as a result of your queer or trans identity? What did you or others learn from these experiences?

I have been fortunate enough to avoid direct challenges in my professional life due to my queer identity. This is largely thanks to a supportive and encouraging work environment, and partly because I wasn’t openly ‘out’ during the early stages of my career, prior to my PhD. However, there are indirect challenges, such as the constant evaluation of how one’s queer or trans identity might be received in various professional contexts. The fear of discrimination causes many who identify as LGBTQ+ to refrain from being open about their identities. This highlights the importance of cultivating truly inclusive and supportive environments that empower everyone to bring their whole selves to their work. Unfortunately, workplace discrimination, a sad part of queer and trans history, persists in some places today.

How can individual scientists support and celebrate their LGBTQ+ colleagues?

To support and celebrate their LGBTQ+ colleagues, they may act as allies. Allyship involves acknowledging diversity, respecting individual identities, and educating themselves about the unique experiences of others. For example, this includes using inclusive language, to convey respect. Allyship doesn’t have to be a passive state. Allies can actively participate in promoting inclusive environments, which includes raising awareness of diversity issues, advocating against discrimination, and promoting equality. It essentially means leveraging personal privilege to support and uplift others.

What action(s) do you feel employers in chemical research should take to make a difference for LGBTQ+ scientists?

Employers in chemical research and in general should cultivate an inclusive culture where diversity is celebrated. This involves enforcing clear anti-discrimination policies and communicating them regularly. Moreover, they should provide platforms for marginalized groups to share their experiences and actively listen to them. It’s also important for employers to identify and address key challenges on this road and track progress over time. The proactive approach can gradually lead to a more welcoming and inclusive environment. Explicit support is important. Utilization of resources like the LGBT+ toolkit from the Royal Society of Chemistry is one example. This toolkit provides strategies for supporting LGBTQ+ individuals in STEM, making it a valuable tool for promoting inclusivity in our field.

How do you lift yourself and potentially the LGBTQ+ community up to thrive in chemical research?

The path to uplift oneself and the LGBTQ+ community to thrive in chemical research and beyond begins with promoting visibility and representation. By presenting accomplished queer role models, we can promote changes in society and institutions and show to the LGBTQ+ community that we can have a secure place in the scientific community too. In doing so, we highlight the accomplishments and contributions of LGBTQ+ individuals within these fields, but also the crucial role they play in advancing scientific knowledge. One of my biggest role models is Lynn Conway. I also look up to many openly out chemists too.

Yet, visibility is just the starting point. There are grassroots initiatives and organizations that work hand-in-hand with academic institutions and corporate entities to further this cause. I am proud to be one of the co-founders of such an initiative, LGBTQ STEM Berlin, and there are many more similar movements around the globe. By providing a platform for queer speakers and fostering networking events, we can create stronger connections between the LGBTQ+ community and the wider scientific ecosystem.

*This interview was conducted by the editors of Communications Chemistry*.
